# Iranian Nurses' Attitudes and Perception towards Patient Advocacy

**DOI:** 10.5402/2012/645828

**Published:** 2012-12-30

**Authors:** Mohadeseh Motamed-Jahromi, Abbas Abbaszadeh, Fariba Borhani, Homa Zaher

**Affiliations:** ^1^Razi School of Nursing and Midwifery, Kerman University of Medical Sciences, Kerman 7616913555, Iran; ^2^School of Nursing and Midwifery, Shahid Beheshti University of Medical Sciences, Tehran 1981619573, Iran

## Abstract

Patient advocacy is an inherent component of professional nursing ethics; in other words, nurses' enough knowledge would be essential to gain a positive attitude towards nursing advocacy. Using a descriptive-analytic design, this study aimed to assess the correlation between nurses' perception and attitudes towards patient advocacy, amongst 385 nurses in Kerman, Iran; hence, a three-part questionnaire was applied: part I, a demographic data sheet, part II, attitude measuring instrument, and part III, perception measuring instrument in nursing advocacy. The results implied that fairly positive attitudes and perception were found amongst the participants, and nurses' attitudes, in general, were positively correlated to their perception toward nursing advocacy. This means that with an improvement in perception, the attitude would also improve. In addition to our findings, it seems that these nurses needed more advocacy educational programs and support from responsible employers.

## 1. Introduction

Patient advocacy is an intrinsic essence of professional nursing ethics. This ethical principle is vital to the nurse-patient relationship and reveals a thought of reverence towards patients as human beings [1] and towards patients' rights [2, 3]. Indeed, patients' needs or desires are recognized as a key impulsion for advocacy in nursing [4]. 

Therefore, it is defined as a crucial section of *nurses' attempts* to encourage and protect health and interests of patients by supplying information and assisting clients in their decisions [5], cooperating to patients' self-determination, autonomy, or empowerment [6], pleading the reason of client [7], defending the client from pointless worries [8], revealing information about misbehavior that imperils the welfare of others [9], and respecting patients values and beliefs, together with educating and interceding [6].

Curtin and Gadow stated that advocacy is the elemental foundation for nursing and exclusively illustrates the essence of nursing practice [10, 11]. Gadow alluded to advocacy as the “philosophical basis and aim of nursing.” Maybe the clearest definition of advocacy in nursing can be found in Gadow's explanation. Advocacy not only safeguards but positively contributes to the exercise of self-determination [11, page 53].

Kohnke recommended that advocacy is a type of caring and kindness in nursing practice and that it is a learned skill that nurses would develop through different experiences, especially if advocacy is esteemed as worthwhile [5]. A great deal of the argument of patient advocacy is originated from the identification and valuing of patient rights and the task of nurses as advocates for the benefits and rights of people [12, 13]. 

These definitions emphasize the nurse's role as a faithful observer of the patient's circumstance [4, 14], signifying an ethical commitment to make certain quality of care [15]. 

It is necessary that nurses comprehend and show their scope of practice and specify their professional autonomy in the health care team, in the organizations, and especially in the community. If nursing professionals want to make their clients powerful, they must empower themselves in the first place [16] and develop the ability to make choices [17], such that the choice or decision on how to deal with patient advocacy would lead to suitable perception and attitudes towards patient advocacy. If the knowledge about patient advocacy is adequate, there would be correct perception leading to positive attitude towards patient advocacy and vice versa. 

Perception is the ability to see, hear, or understand a certain event and apply assumptions about the world's arrangement to integrate sensory information [18]. An attitude refers to feelings, beliefs, and positive and negative reactions of an individual towards an event, phenomenon, object, or person to arise through the internalization of cognitive structures [19].

The literature contained convincing arguments in favor of nurses' assumption regarding the role of patient advocate. Nurses' intrinsic characteristics are cited as critical components in nurses' ability to act as nursing advocates [20, 21]. It seems that nurses' characteristics such as self-concept, personal values, confidence as nurses, and personal beliefs play a main role in nurses' advocacy actions. Generally, only a few quantitative studies have evaluated nursing advocacy, and the majority have been qualitative [22]. In Iran, Negarandeh et al. have reported one qualitative grounded theory-type study to inquire into the meaning of patient advocacy from Iranian nurses' perspective [23]. According to the *investigators*, no research with quantitative method, to measure nurses' attitudes and perception relationship, was found in nursing advocacy in Iran and other countries. So, this descriptive-analytic study aimed to examine nurses' attitudes and perception towards patient advocacy in an Iranian health care context with a quantitative method; this may help to clarify the meaning of this complex issue.

## 2. Methods

### 2.1. Design

The proposal of this study was approved in Kerman Razi School of Nursing and Midwifery. The research approval and ethic committees of Kerman University of Medical Sciences approved this study, and the permission to conduct this study was also obtained from the presidents of four educational hospitals in Kerman city. Moreover, a descriptive-analytic design was applied, and this study was conducted in those four educational hospitals. Informed consent was obtained from all participants, and they were free to decline to participate or to consent but later withdraw their consent without prejudicing their position in the organization.

### 2.2. The Instruments

A comprehensive three-part questionnaire was applied to describe the population and collect information regarding nurses' attitudes and perception towards nursing advocacy. Part I, created by us, consisted of one page with seven demographic variables including the hospital name, the ward, age, sex, marital status, nursing work experience, and history of education regarding Patient Right's Workshop. In part II, we designed a questionnaire using selective parts of attitude measuring instruments in nursing advocacy that were formerly developed in two other studies by Barrett-Sheridan [24] and Hanks [25]. The participants in this study were Persian nurses, so those questionnaires were translated into Persian using translation and back-translation techniques by two specialists, and cross-cultured adaptation was also conducted.

At first, the questionnaire was designed with twenty-three questions, and then the number of questions was reduced to nineteen, after factor analysis. The questions were scored from 1 to 5 using a five-point Likert scale (1 = strongly disagree to 5 = strongly agree). Nine items were worded positively and ten were worded negatively. 

A factor analysis was performed in the Iranian setting to divide questions. The data was assessed as factorable using Kaiser Meyer Olkin (KMO) test of “sampling adequacy,” .85, and Bartlett's test of sphericity, *P*  value = 0.00, for factor analysis. So, factor analysis was conducted using principal components analysis (PCA) with varimax rotation scheme. Examination of the eigenvalues and scree plot indicated two factors. The two factors were labeled as factor 1 for cognitive (believe) aspect of attitude (9 items) and factor 2 for behavior (efficacy) aspect of attitude (10 items). 

Part III, the nineteen-item self-administered perception questionnaire, was built by means of diversity studies. The respondents were required to choose their answers from a set of preprovided answers (“yes,” “no,” and “do not know”). The average score for each question was identified in attitude questionnaire with a sorting index of (1-2) negative attitude, (2-3) relatively negative attitude, (3) neutral attitude, (3-4) relatively positive attitude, and (4-5) positive attitude and in perception questionnaire with a sorting index of (0–0.25) weak, (0.25–0.5) relatively weak perception, (0.5–0.75) relatively suitable perception, and (0.75–1) suitable perception.

### 2.3. Reliability and Validity

The validity and reliability of perception and Persian-modified attitude scales were checked. The validity of the scales was assessed through a content validity discussion. An expert panel on nursing ethical issues have reviewed the content of the scales from ethical and cultural aspects of nursing advocacy and agreed on a reasonable content validity. To reassess the reliability of the scales, Cronbach alpha was calculated to evaluate internal consistency among items. Firstly, the alpha coefficient for attitude scale was 0.75, and after factor analysis, this was 0.77, and for questions in factor 1 of attitude scale, it was 0.88, and factor 2 was 0.77. The alpha coefficient for perception scales was 0.87.

### 2.4. Data Collection and Analysis

The written information about the aims of this study was given to the participants in the form of a letter; the questionnaires were handed out by the author to 385 nurses who were selected by quota sampling in four Kerman educational hospitals. The necessary information about the study was orally presented to the participants. Moreover, participation in the study was both voluntary and anonymous. Three hundred seventy-four sets of questionnaires were distributed, with a dropout of eleven. In whole, more than 97% of all questions were answered. Data was analyzed using Statistical Package Social Sciences (SPSS) version 18. The descriptive statistics of the samples and measures included frequencies, means, and reliability. One-way analysis of variance (ANOVA) was applied to examine the relationships between the mean level of attitude, perception, and demographic variables. Pearson correlation analysis was used to show any significant correlation between nurses' attitude and perception.

## 3. Results

### 3.1. Participants

 A descriptive analysis of the demographic information (Table 1) showed that the participants' age ranged from 22 to 55 years with a mean age of 30 years and were mostly females (93%), married (63%), with the Bachelor degree of Science in nursing (98%), with six-month to ten-year experience of working in hospitals (70%). However 54% of the participants were general ward's nurses, and only 37.2% claimed that they were educated patient rights.

### 3.2. Descriptive Findings

The descriptive analysis (Table 2) indicated a fairly positive attitude (mean = 3.79) and perception (mean = 0.783) towards nursing advocacy amongst the participants. It also indicated that the participants had more positive attitude (4.27) toward cognitive aspects compared to behavioral aspect (3.24).

The comparison of the mean scores of the nurses' attitude and perception toward nursing advocacy and demographic characteristics of the nurses (Table 1) revealed that the mean score of attitude and perception amongst the male participants with the Masters degree in nursing, and those who had already participated in Patient Right's Workshop and nurses who were working in psychotic wards, was more than others. In addition, there was a significant difference between the mean scores of attitude (*P*  value = 0.00) and perception (*P*  value = 0.009) of nurses who were working in different hospital.

### 3.3. Correlation Analysis

Pearson correlation analysis (Table 3) showed a significant and positive correlation between nurses' attitude and perception (*r* = .351) toward nursing advocacy and also between cognitive (believe) aspects of attitude (*r* = .497) and behavior (efficacy) aspects of attitude (*r* = .024) with nurses' perception toward nursing advocacy. Based on these results, it seems that the correlation of nurses' perception with the cognitive aspect of attitude is more positive than the behavioral aspect.

According to the figures presented in Table 4, gender and marital status were positively correlated with attitudes, and such a correlation was more positive amongst male and single participants.

It should be noted that the correlation between the participants' attitude and perception and their educational level and the background experience of nursing was also positive, and such a correlation regarding the Bachelor's degree and experience of nursing between twenty to thirty years was more positive when compared to others. The nurses' perception and attitudes amongst those working in psychiatric hospital and psychiatric wards were positively correlated with each other, and this correlation among participating nurses in Patient Right's Workshop was positive too.

In general, according to the results of this study, improving nurses' perception would lead to a promotion in their attitude toward nursing advocacy.

## 4. Discussion

The results of this study indicated that nurses' attitudes and perception toward patient advocacy were fairly positive. These results supported the results of two other studies by Barrett-Sheridan who had found positive nurses' attitude toward nursing advocacy [24] and by Thacker who had stated that most participants agreed with perceptual behavior of nursing advocacy [26].

Furthermore, other findings have shown that nurses' attitudes were positively correlated to their perception (*r* = .351) toward nursing advocacy. This indicates that those who had more positive attitude had also more positive perception. Since perception is the ability to see, hear, or understand a certain event and awareness to improve upon such power of perception [27] and attitudes are acquisitive responses that allude to feelings, values, and reactions of an individual towards an occurrence phenomena [28], if nurses' knowledge toward nursing advocacy is not acceptable enough, it will be perceived wrongly or negatively, and this can lead to a negative or neutral attitude towards nursing advocacy. If the knowledge about nursing advocacy is sufficient and understandable, correct or right perception would be gained, and this would lead to a positive attitude towards nursing advocacy.

The authors of this study concluded that the nurses who were working in psychiatric wards of psychiatric hospitals had more positive attitude and perception compared to others; it seems that these nurses' knowledge and perception of nursing advocacy is more than others, and they put this knowledge into practice, indicating their positive attitude. According to Boutell and Bozett, mental health nurses were more likely to evaluate patient's spiritual needs because they had more time and were accustomed to consult patients [29].

## 5. Limitations

This study was exclusively performed in the city of Kerman, Iran. So, there is a need to conduct more studies in vaster areas and to increase the sample size for improving generalization. It is better to carry out other studies with a larger sample size in more cities.

## 6. Conclusion

The present study with a focus on Iranian nurses' attitudes and perception towards the responsibilities of patient advocacy explains how nurses' positive perception would influence on increasing nurses' positive attitude. It goes without saying that the Iranian nurses have been advocated for patients since many years ago, without having nursing advocacy as a professional role in Iran, advocacy educational programs, and supports from their employers for doing this role. Furthermore, educating nursing advocacy is necessary in the Iranian nursing student curriculum, and it should be continued for nurses to improve the quality of this role. More studies will brightly develop the identification of advocate role and support changes needed in the workplace setting to promote advocacy action for patients' sake.

## Figures and Tables

**Table 1:** Demographic data.

Background characteristics	*n*	%	Attitude mean	*P* value	Perception mean	*P* value
Age (year)				0.00		0.213
<30	184	49.2	3.68		0.75	
30–40 y	151	40.4	3.89		0.74	
>40	27	7.2	3.93		0.64	
Hospital				*0.00 *		*0.009 *
Hospital no. 1	130	34.8	3.72		0.70	
Hospital no. 2	111	29.7	3.69		0.68	
Hospital no. 3 (psychiatric)	28	7.5	*4.17 *		*0.81 *	
Hospital no. 4	105	28.1	3.86		0.80	
Gender				0.04		0.230
Female	350	93.6	3.77		0.73	
Male	24	6.4	*3.99 *		*0.80 *	
Marriage				0.14		0.501
Single	138	36.9	3.74		0.75	
Married	235	62.8	3.81		0.73	
Education				0.65		0.748
B.S.	369	98.7	3.78		0.73	
M.S.	5	1.3	*3.88 *		*0.77 *	
Patient Right's Workshop				0.00		0.081
Yes	139	37.2	*3.99 *		*0.77 *	
No	235	62.8	3.66		0.71	
Nursing work experience (year)				0.03		0.132
6 m–10 y	262	70.1	3.72		0.74	
10–20 y	89	23.8	3.94		0.73	
20–30 y	18	4.8	3.89		0.59	
Ward				0.00		0.319
Critical wards	142	38	3.79		0.73	
General wards	204	54.4	3.73		0.72	
Mental wards	28	7.5	*4.17 *		*0.81 *	

**Table 2:** 

Total attitude mean	3.79	Relatively positive
Cognitive (believe) aspect of attitude mean	4.27	Positive
Behavior (efficacy) aspect of attitude mean	3.24	Relatively positive

Total perception mean	0.73	Relatively suitable

**Table 3:** Attitude and perception correlation.

Pearson correlation	Perception mean
Attitude mean	*r* = .351*
Factor 1 = cognitive (believe) aspect of attitude	*r* = .497*
Factor 2 = behavior (efficacy) aspect of attitude	*r* = .024*

*Correlation is significant at the level of *P* < .05.

**Table 4:** Attitude, perception, and demographic factor correlation.

Pearson correlation	Attitude and perception correlation	
Demographic factor	Age (year)	
	<30	*r* = .365*
	30–40 y	*r* = .331*
	>40	*r* = .575*
	Gender	
	Female	*r* = .326*
	Male	*r* = .532*
	Marriage	
	Single	*r* = .389*
	Married	*r* = .352*
	Education	
	B.S.	*r* = .352*
	M.S.	*r* = .224*
	Patient Right's Workshop	*r* = .309*
	Nursing work experience (year)	
	6 m–10 y	*r* = .397*
	10–20 y	*r* = .193*
	20–30 y	*r* = .643*
	Ward	
	Critical wards	*r* = .327*
	General wards	*r* = .315*
	Mental wards	*r* = .652*
	Hospital	
	No. 1	*r* = .411*
	No. 2	*r* = .232*
	No. 3 (psychiatric)	*r* = .652*
	No. 4	*r* = .008*

^∗^Correlation is significant at the level of *P* < 0.05.

## References

[1] Hem MH, Heggen K (2004). Is compassion essential to nursing practice?. *Contemporary Nurse*.

[2] Snowball J (1996). Asking nurses about advocating for patients: reactive and proactive accounts. *Journal of Advanced Nursing*.

[3] Webb C (1987). The nurse advocate: speaking up for advocacy. *Nursing Times*.

[4] Schroeter K (2000). Advocacy in perioperative nursing practice. *AORN Journal*.

[5] Kohnke MF (1982). Advocacy: risk and reality. *Mosby*.

[6] Baldwin MA (2003). Patient advocacy: a concept analysis. *Nursing Standard*.

[7] Mitty EL (1991). The nurse as advocate. Issues in LTC. *Nursing & Health Care*.

[8] Malin N, Teasdale K (1991). Caring versus empowerment: considerations for nursing practice. *Journal of Advanced Nursing*.

[9] McDonald S, Ahern K (2000). The professional consequences of whistleblowing by nurses. *Journal of Professional Nursing*.

[10] Curtin IH (1979). The nurse as advocate: a philosophical foundation for nursing. *Advances in Nursing Science*.

[11] Gadow S (1990). Existential advocacy: philosophical foundations of nursing. *NLN Publications*.

[12] Bandman EL, Bandman B (2002). *Nursing Ethics Through the Life Span*.

[13] Mallik M (1997). Advocacy in nursing—a review of the literature. *Journal of Advanced Nursing*.

[14] Hyland D (2002). An exploration of the relationship between patient autonomy and patient advocacy: implications for nursing practice. *Nursing Ethics*.

[15] Vaartio H (2008). *Nursing advocacy: a concept clarification in context of procedural pain care [Doctoral dissertation]*.

[16] Ryles SM (1999). A concept analysis of empowerment: its relationship to mental health nursing. *Journal of Advanced Nursing*.

[17] Kuokkanen L, Leino-Kilpi H (2000). Power and empowerment in nursing: three theoretical approaches. *Journal of Advanced Nursing*.

[18] Smith EE, Nolen-Hocksema S (2004). *Atkinson and Hilgards's Introduction to Psychology*.

[19] Rital A (1975). *Introduction to Psychology*.

[20] Hinson Penticuff J (1989). Infant suffering and nurse advocacy in neonatal intensive care. *Nursing Clinics of North America*.

[21] Sellin SC (1995). Out on a limb: a qualitative study of patient advocacy in institutional nursing. *Nursing Ethics*.

[22] Hanks RG (2010). The medical-surgical nurse perspective of advocate role. *Nursing Forum*.

[23] Negarandeh R, Oskouie F, Ahmadi F, Nikravesh M (2008). The meaning of patient advocacy for Iranian nurses. *Nursing Ethics*.

[24] Barrett-Sheridan SE (2009). *A Quantitative Correlational Study of Political Behavior and Attitudes of Nurses Toward Macro-Social Patient Advocacy*.

[25] Hanks RG Protective Nursing Advocacy Scale.

[26] Thacker KS (2008). Nurses’ advocacy behaviors in end-of-life nursing care. *Nursing Ethics*.

[27] Adewuyi TDO, Akinade EA Perception and attitudes of Nigerian women towards menopause.

[28] Adewuyi TDO *Effect of rational emotive behavioural and reality therapies on attitude of federal teachers towards retirement [Unpublished Ph.D. thesis]*.

[29] Boutell KA, Bozett FW (1990). Nurses’ assessment of patients’ spirituality: continuing education implications. *Journal of Continuing Education in Nursing*.

